# Effects of internal migration on healthcare services utilization in Bangladesh: an analysis of nationally representative survey

**DOI:** 10.3389/fpubh.2025.1495977

**Published:** 2025-06-04

**Authors:** Fardin Araf, Md Rabiul Haque, Gustavo Angeles

**Affiliations:** ^1^Department of Population Sciences, Faculty of Social Science, University of Dhaka, Dhaka, Bangladesh; ^2^Data for Impact (D4I), Carolina Population Center, University of North Carolina at Chapel Hill, Chapel Hill, NC, United States

**Keywords:** antenatal care, internal migration, delivery care, migration and maternal healthcare, Bangladesh

## Abstract

**Background:**

Despite the significance of internal migration as an important social determinant of health that could potentially affect the utilization of maternal healthcare services, the magnitude of this relationship by different migration streams is yet to be fully explored in Bangladesh.

**Methods:**

This study using Bangladesh Demographic and Health Survey data, 2017–18 examined the effects of different migration streams on maternal healthcare service utilization, particularly four or more antenatal care (≥4 ANC) visits and institutional delivery (ID) care services.

**Results:**

The analysis identified significant variations in using antenatal and institutional delivery care services between migrants and non-migrants. The rural non-migrants were found to be the most disadvantaged group, particularly when different forms of migration streams were considered. For instance, after adjusting for covariates, urban to urban migrants (≥4 ANC = 1.866, *p* < 0.01; ID = 2.247, *p* < 0.001) and urban to rural migrants (≥ 4 ANC = 1.24, *p* > 0.05; ID = 1.689, *p* < 0.05) were more likely to utilize both types of maternal healthcare services than rural non-migrants. However, migrants of all streams were less likely to use any type of maternal healthcare services when compared against the urban non-migrants.

**Conclusion:**

Addressing the effects of migration in designing and implementing maternal healthcare service delivery programs may address the needs and challenges faced by migrants.

## 1 Introduction

Being an important social determinant of health, change of residence or migration has long received attention in both public health and the social sciences with gradually increased emphasis on the link between migration and the utilization of sexual, reproductive, maternal, and child healthcare services (1). The existing evidence indicates the intertwined and complex relationships between population movement and healthcare (2). The decision to migrate can be influenced by health considerations, while the migration experience itself can have significant impacts on the health outcomes of migrants, as well as those who remain in the place of origin and even those who host the migrants in the destination location. Studying healthcare in the context of migration provides a comprehensive understanding of the intricate and heterogeneous nature of the migration process (3). With the surge in migratory movements globally, the health needs and well-being of migrants have become a pressing issue that demands more attention (4, 5).

Moving to a new residence can have a direct impact on an individual’s health, which can either improve or deteriorate health status depending on various factors including changes in healthcare accessibility, exposure to new environments, lifestyle adjustments, and sociocultural differences. Among migrants, females are often considered more vulnerable in terms of reproductive health issues compared to their male counterparts (6, 7). The absence of sexual, reproductive, maternal, and child healthcare services has been cited as one of the top three health issues confronted by internal migrants, alongside communicable and occupational diseases (5, 8). Migrant women in Bangladesh, like many other countries, also encounter multiple challenges in accessing and utilizing adequate maternal healthcare services (3).

The access to and utilization of maternal healthcare services among Bangladeshi women differ by several factors such as place of residence, lack of women’s education, women and husband’s occupation, household poverty, higher birth order, women’s attitude toward domestic violence, geographical obstacles, climate displacement, internal migration, etc. (9–11). Although national-level data from the Bangladesh Demographic and Health Survey and Urban Health Survey indicate remarkable progress in maternal health indicators, disparities in access and utilization were found to be particularly prevalent in urban areas, characterized by high levels of poverty, inequality, and inadequate infrastructure (12, 13). With 40.5% of the total population living in urban areas—a figure expected to double by 2050, and internal migration reportedly accounting for 66% of this urban growth, the role of internal migration in urban growth and its implications for maternal healthcare service utilization (MHCSU) becomes even more critical to understand (14–16).

This issue is particularly critical given the disparities in MHCSU among different population groups, with migrants potentially facing unique challenges. Current literature indicates variations in MHCSU between migrants and non-migrants, particularly in ≥4 ANC and ID in the context of Bangladesh and India (9, 11, 12, 17–20). Nonetheless, these insights are often limited as they concentrated predominantly on urban areas and small sample sizes and were not nationally representative. Furthermore, these studies generally did not comprehensively explore how different types of internal migration streams influence MHCSU. Our study aims to fill this research gap by examining the effect of various internal migration streams on ≥4 ANC and ID service utilization, as recommended by WHO (21). After controlling for individual and household characteristics using multivariate regression analysis, we specifically aimed to ascertain whether women who participated in various migration pathways (rural-to-rural, rural-to-urban, urban-to-rural, and urban-to-urban) showed distinct patterns of maternal healthcare use in comparison to non-migrant women in both rural and urban settings.

## 2 Methodology

### 2.1 Data source and sample population

Data for this study were extracted from the Bangladesh Demographic and Health Survey 2017–18, a national-level survey (13). After a request explaining the purpose, permission to use the data was formally approved.^1^ In line with the study objectives, women aged 15–49 years who gave birth in the last 3 years and sought maternal healthcare services after internal migration and non-migrant women aged 15–49 years were selected for analysis.

The DHS surveys collect comprehensive birth histories from interviewed women but gather the most detailed information on maternal care (antenatal, delivery, postnatal), newborn care, child health (including vaccinations and recent illnesses), and related topics specifically for births occurring within a recent window – typically the 3 years preceding the survey date. This study also focused the analysis on births 3 years prior to the BDHS 2017–18 survey The total number of women who gave birth in the last 3 years was 5,012. Of these, 651 visitors’ women were excluded from the analysis, reducing the sample size to 4,361. Furthermore, those who are currently internal migrants, but had sought maternal healthcare services before their migration (392), were also excluded, which left a final sample size of 3,969 (unweighted). The eligible sample was further divided into two categories, non-migrants = 539; and migrants = 3,430. After all the exclusion criteria were applied, the weighted number of migrants and non-migrants was 3,460 and 554 respectively, resulting in a final weighted sample size of 4,014 women aged 15–49.

### 2.2 Dependent variables

This study examined two primary dependent variables related to maternal healthcare utilization. First, antenatal care (ANC) utilization was assessed based on the number of ANC services received during the respondent’s last pregnancy: women receiving four or more visits were categorized as ‘adequate ANC users,’ while those receiving fewer than four were classified as ‘inadequate ANC users.’ Second, the place of delivery for the most recent live birth within the 3 years preceding the survey was investigated. This variable was dichotomized into ‘home delivery’ and ‘institutional delivery’ (delivery in a health facility).

### 2.3 Independent variable

The primary independent variable for this study was the internal migration stream. Participants were initially classified as ‘non-migrants’ if they had always lived in their current residence, or as ‘migrants’ if they provided a specific duration of stay at their current residence. Following this, the migration stream for each participant was classified based on a comparison of their previous and current residence types (rural/urban). This classification led to six mutually exclusive categories: rural non-migrant, urban non-migrant, rural-to-rural (r-r) migrant, rural-to-urban (r-u) migrant, urban-to-urban (u-u) migrant, and urban-to-rural (u-r) migrant. The non-migrant categories served as reference groups in the analysis.

### 2.4 Study covariates

An extensive literature review was conducted to identify potential covariates associated with maternal healthcare service utilization. Based on this review and data availability in the BDHS 2017–18 dataset, this study included a total of 16 covariates. These were: women’s age, education, working status, autonomy, age at marriage, household wealth index, sex of household head, household size, religion, number of living children, child desirability status, husband’s education, occupation, women’s awareness of community clinic nearby her vicinity, women’s healthcare barriers (whether distance and money for treatment is a problem for women or not), and women’s mass media exposure. In this study, the BDHS constructed wealth index was used which is a composite measure living standard of a household constructed using household assets data.

### 2.5 Data analysis

Descriptive statistics such as frequency, and percentage (for categorical data) were considered in the univariate stage. At the bivariate stage, the chi-square test was conducted with a 5% level of significance.

Binary logistic regression with the backward L.R. method was applied at the multivariate level. Since the outcome variable is dichotomous, binary logistic regression was used. Three regression models were developed for the analysis. All three binary logistic regression models used odds ratio and 95% confidence intervals to draw statistical conclusions. In model 1, we performed a simple logistic regression to examine the relationship between the dependent variable and the key independent variable, migration stream, along with study covariates. In model 2, migration selection-related variables were controlled. Variables entered in Model 2 were respondent’s age, education, working status, wealth, and women’s autonomy. The migrant selection theory suggests that certain variables included in the model contribute to the differences between migrant and non-migrant populations. We controlled migration selection-related variables to compare the different characteristics of migrants and non-migrants, aiming to analyze the distinct effects of migration on MHCSU (22).

Finally, in model 3, all the variables known to affect maternal healthcare utilization were entered into the analysis. Variables entered in model 3 were sex of the household head, household size, religion, women’s age at marriage, child desirability status, number of living children, husband’s occupation and education, barriers to reaching health facility (distance and money), and women’s mass media exposure. By doing so, this study controlled the determinants of maternal healthcare service utilization and adjusted for the effect of all the potential confounders in migration and maternal healthcare-seeking relationships. The final model allowed us to examine the strength of the relationship between migration and MHCSU even after controlling the effects of other determinants of MHCSU.

## 3 Results

Table 1 presents the background characteristics of female internal migrants and non-migrants aged 15–49 who had given birth in the last 3 years. The weighted sample population included a total of 3,453 (86.1%) migrants and 554 (13.8%) non-migrants. The highest proportion of women possessed secondary education (49.4%), followed by primary education (28.7%). The proportion of women with more than secondary education was notably higher than women with no education at all. Regarding working status, the majority of women were not working (57.8%), while the rest were employed (42.2%). The study population was relatively evenly distributed across the wealth quintiles. The largest proportion of women fell within the “poorest” category (21.8%), followed by the “poorer” category (20.5%). The “middle” category (19.8%) had the third-largest proportion of women, followed by the “richer” category (19.5%) and the “richest” category (18.4%).

**Table 1:** Distribution of women aged 15–49 years by demographic and socioeconomic attributes.

Background characteristics	Weighted *n*, (%)
Migratory status	
Migrant	3,453 (86.1)
Non-migrant	554 (13.8)
Age of the respondents	
15–19	662 (16.50)
20–29	2,412 (60.10)
30–39	892 (22.20)
40–49	48 (1.20)
Women’s educational level	
No education	268 (6.70)
Primary	1,153 (28.70)
Secondary	1985 (49.40)
Higher	609 (15.20)
Women’s working status	
Not working	2,320 (57.80)
Working	1,692 (42.20)
Wealth index	
Poorest	874 (21.80)
Poorer	823 (20.50)
Middle	795 (19.80)
Richer	784 (19.50)
Richest	739 (18.40)
Sex of household head	
Male	3,506 (87.30)
Female	508 (12.70)
Household size	
3 members	499 (12.40)
4 members	844 (21.00)
5 members	847 (21.10)
6 &7 members	1,053 (26.20)
8 and above	771 (19.20)
Religion	
Islam	3,686 (91.80)
Others	328 (8.20)
Age at marriage	
Married before 18	2,870 (71.50)
Married at age 18 and above	1,144 (28.50)
Number of living children	
Two and less	2,841 (71.00)
More than two	1,163 (29.00)
Child desirability status	
Wanted	3,161 (78.70)
Mistimed	499 (12.40)
Unwanted	354 (8.80)
Husband education	
No education	595 (15.00)
Primary	1,430 (35.90)
Secondary	1,311 (32.90)
Higher	643 (16.20)
Husband occupation	
Not working	71 (1.80)
Professional, technical, managerial	295 (7.40)
Sales, service, and manual worker (industrial)	2,801 (69.90)
Agricultural, domestic, and unskilled manual worker	839 (20.90)
Aware of any community clinic	
No	1,547 (38.50)
Yes	2,467 (61.50)
Healthcare barriers (distance & money)	
Yes	1,044 (26.00)
No	2,970 (74.00)
Mass media exposure	
Not exposed	1,473 (36.70)
Exposed	2,541 (63.30)
Women’s autonomy	
Low autonomy	1,119 (28.10)
Moderate autonomy	2,566 (64.50)
High autonomy	293 (7.40)
Place of delivery	
Home delivery	2074 (51.7)
Institutional delivery	1940 (48.3)
ANC utilization	
Less than 4 ANC	2,175 (54.2)
4 and above	1839 (45.8)

As far as awareness regarding community clinics was concerned, 61.5% of the individuals were aware of any community clinic in their area. Besides, the table indicated that accessibility to healthcare was problem for some individuals in the study population. Approximately 26.0% of the women reported ‘yes’ to distance and money for treatment being an issue to access healthcare. Furthermore, the data showed that 63.3% of the women were exposed to mass media. The table also presents information on the level of women’s autonomy in household decision-making. The table indicates that 64.5% of women reported moderate autonomy in household decision-making, while 28.1% reported low autonomy, and 7.20% reported high autonomy. In terms of place of delivery, 51.7% of women had delivered birth at home, whereas 48.3% delivered at a health center. Besides, 54.2% of women had utilized less than 4 ANC and 45.8% have utilized four or more ANC.

### 3.1 Distribution of women by migration streams

Table 2 presents the distribution of the weighted sample population by migration status. The most dominant stream was rural-to-rural migration, accounting for 60.33% of the sample. This was followed by migration from rural to urban areas (15.22%). The least frequent migration stream occurred from urban to rural areas (4.14%).

**Table 2:** Study population by migration status.

Migration streams	*N*	%
Rural non-migrant	404	10.08
Rural to rural	2,418	60.33
Urban to rural	166	4.14
Urban to urban	260	6.49
Rural to urban	610	15.22
Urban non-migrant	150	3.74

### 3.2 Bivariate findings

The bivariate findings presented in Table 3 suggested significant variations in maternal healthcare utilization across migration streams. Rural non-migrants had the lowest prevalence (38.9%) of utilizing ≥4 ANC visits, closely followed by rural-to-rural migrants (40.8%). The prevalence was notably higher for urban-to-rural migrants (53.9%). Urban-to-urban migrants had the highest prevalence of adequate ANC utilization (70.3%), followed by urban non-migrants (69.3%). Rural-to-urban migrants also had a higher prevalence of adequate ANC use (51.8%) compared to rural non-migrants and rural-to-rural migrants.

**Table 3:** Prevalence of receiving 4 and more ANC and institutional delivery care by socio-economic characteristics.

Migration stream	% of ≥4 ANC	*p*-Value	% of ID	*p*-Value
Rural non-migrant	38.90	0.000	37.60	0.000
Rural to rural	40.80		43.10	
Urban to rural	53.90		59.60	
Urban to urban	70.30		77.30	
Rural to urban	51.80		54.80	
Urban non-migrant	69.30		72.70	
Age of the respondents				
15–19	46.80	0.358	49.70	0.206
20–29	46.50		49.10	
30–39	43.20		45.60	
40–49	45.80		41.70	
Women’s educational level				
No education	19.40	0.000	25.70	0.000
Primary	34.00		32.40	
Secondary	48.80		51.50	
Higher	70.30		78.30	
Women’s working status				
Not working	46.70	0.159	54.10	0.000
Working	44.50		40.30	
Wealth index				
Poorest	29.70	0.000	26.10	0.000
Poorer	35.00		34.60	
Middle	45.80		47.40	
Richer	50.60		59.80	
Richest	71.80		78.90	
Sex of household head				
Male	46.40	0.067	48.60	0.363
Female	42.00		46.50	
Household size				
3 members	48.10	0.087	54.50	0.023
4 members	47.90		48.50	
5 members	45.20		46.80	
6 &7 members	42.50		45.90	
8 and above	47.30		49.30	
Religion				
Islam	44.90	0.000	47.30	0.000
Others	55.70		60.20	
Age at marriage				
Married before 18	42.60	0.000	43.20	0.000
Married at age 18 and above	54.00		61.30	
Number of living children				
Two and less	50.90	0.000	54.90	0.000
More than two	33.50		32.50	
Child desirability status				
Wanted then	48.00	0.000	50.90	0.000
Wanted later	43.40		44.70	
Wanted no more	29.70		30.90	
Husband education				
No education	28.70	0.000	29.40	0.000
Primary	37.20		38.00	
Secondary	51.20		54.10	
Higher	70.50		77.90	
Husband occupation				
Not working	35.20	0.000	47.90	0.000
Professional, technical, managerial	71.90		76.30	
Sales, service, and manual worker	46.20		49.50	
Agricultural, domestic, and unskilled manual worker	36.20		34.20	
Aware of any community clinic				
No	50.50	0.000	53.20	0.000
Yes	42.90		45.30	
Healthcare barriers (distance & money)				
Yes	35.50	0.000	36.30	0.000
No	49.50		52.60	
Mass media exposure				
Not exposed	31.60	0.000	31.70	0.000
Exposed	54.10		58.00	
Women’s autonomy				
Low autonomy	46.10	0.991	48.90	0.015
Moderate autonomy	45.90		47.40	
High autonomy	45.70		56.30	

Regarding institutional delivery, the findings suggested that rural non-migrants were in the most disadvantageous position, with only 37.6% delivering in a health facility. Those who migrated from rural-to-rural areas and rural-to-urban areas fared better, with institutional delivery prevalence rates of 43.1 and 54.8%, respectively. The prevalence among urban-to-urban migrants was the highest across all streams (77.3%). The results of the chi-square tests indicated that these observed differences in both ≥4 ANC use and institutional delivery across migration streams were statistically significant (*p* < 0.001).

### 3.3 Multivariate findings

#### 3.3.1 Effect of migration streams on four or more ANC service utilization

The results of binary logistic regressions examining the effect of migration streams on at least 4 ANC utilization are presented in Table 4. Model 1 included only the migration stream variable. Model 2 controlled five potential migrant selection factors (respondent’s age, education, working status, wealth, and autonomy). As suggested by migrant selection theory, the adjusted results from model 2 will provide the sole effect of migration on ANC service and institutional delivery care utilization. In model 3, a total of 11 additional study covariates are added that are known from the literature to affect maternal healthcare services utilization to adjust for the potential confounders in migration and maternal healthcare-seeking relationship and to see if the relationship is strong enough to exist when controlled for all the variables at once. The results suggested that migration streams had a significant effect on ≥4 ANC utilization across the models, although the strength and significance varied by stream and model specification.

**Table 4:** Effect of migration streams on ≥ 4 ANC service utilization.

Migration variables	OR, 95%CI, Model 1	AOR, 95%CI, Model 2	AOR, 95%CI, Model 3
Rural non-migrant	Reference	Reference	Reference
Rural to rural	1.08 (0.87–1.34)	0.99 (0.79–1.24)	0.98 (0.77–1.24)
Urban to rural	1.84** (1.28–2.65)	1.24 (0.84–1.82)	1.11 (0.75–1.65)
Urban to urban	3.71*** (2.66–5.17)	1.86** (1.28–2.70)	1.82** (1.24–2.65)
Rural to urban	1.69*** (1.31–2.18)	1.17 (0.88–1.54)	1.17 (0.88–1.56)
Urban non-migrant	3.57*** (2.39–5.33)	2.14** (1.37–3.35)	2.16** (1.37–141)
Nagelkerke R-square	0.047	0.158	0.192

**p < 0.01, ***p < 0.001; Backward L.R Logistic Regression Method is Employed; Model 2 is adjusted for Respondent’s Age, Education, Working Status, Wealth and Women’s Autonomy; Model 3 is adjusted for Sex of the Household Head, Household Size, Religion, Marriage Age, Child desirability status, Number of Living children, Husband Occupation, Husband Education, Healthcare Accessibility Problem, Mass Media Exposure.

Model 1 (unadjusted), a simple logistic regression, found that compared to rural non-migrants, the odds of using ≥4 ANC are higher for various migration streams: urban–rural (OR = 1.845), urban–urban (OR = 3.711), rural–urban (OR = 1.695), and urban non-migrant (OR = 3.575). After adjusting for selection factors in Model 2, only urban-to-urban migrants (AOR = 1.866) and urban non-migrants (AOR = 2.147) showed significantly increased odds compared to rural non-migrants; the effects for other migration streams became statistically insignificant. In the fully adjusted Model 3, only urban-to-urban migrants (AOR = 1.825) and urban non-migrants (AOR = 2.164) maintained significantly higher odds of receiving ≥4 ANC visits compared to rural non-migrants. The effects for other migration streams were not statistically significant in Model 3.

#### 3.3.2 Effect of migration streams on institutional delivery care

Similar regression models were run for institutional delivery, with results presented in Table 5. These results also suggested that migration streams had a significant effect on institutional delivery utilization, although effects attenuated across models. Model 1, highlighted that all migration streams showed a significantly higher likelihood of institutional delivery compared to rural non-migrants, with urban-to-urban migrants having the highest odds (OR = 5.684).

**Table 5:** Effect of migration streams on institutional delivery care.

Migration variables	OR, 95%CI, Model 1	AOR, 95%CI, Model 2	AOR, 95%CI, Model 3
Rural non-migrant	Reference	Reference	Reference
Rural to Rural	1.25* (1.00–1.55)	1.19 (0.94–1.18)	1.18 (0.92–1.51)
Urban to Rural	2.46*** (1.70–3.56)	1.68* (1.12–2.541)	1.51 (0.99–2.30)
Urban to Urban	5.68*** (3.99–8.09)	2.24*** (1.50–3.347)	2.09*** (1.38–3.16)
Rural to Urban	2.00*** (1.55–2.59)	1.23 (0.92–1.648)	1.2 (0.88–1.62)
Urban non-migrant	4.33*** (2.87–6.54)	2.21** (1.38–3.557)	2.06** (1.26–3.37)
Nagelkerke R Square	0.063	0.258	0.278

*p < 0.05, **p < 0.01, ***p < 0.001; Backward L.R Logistic Regression Method is Employed; Model 2 is adjusted for Respondent’s Age, Education, Working Status, Wealth and Women’s Autonomy; Model 3 is adjusted for Sex of the Household Head, Household Size, Religion, Marriage Age, Child desirability status, Number of Living children, Husband Occupation, Husband Education, Healthcare Accessibility Problem, Mass Media Exposure.

Model 2, which introduced controls for migrant selection factors, showed reduced odds ratios, but urban-to-rural (AOR = 1.689), urban-to-urban (AOR = 2.247), and urban non-migrant (AOR = 2.210) groups remained significantly more likely than rural non-migrants to use institutional delivery. In the fully adjusted Model 3, only urban-to-urban migrants (AOR = 2.090) and urban non-migrants (AOR = 2.069) exhibited significantly higher odds of institutional delivery compared to rural non-migrants; other migration streams lost statistical significance in this final model.

## 4 Discussion

This is one of the early studies in the context of Bangladesh that has operationalized multiple migration-related variables aimed at an extensive comprehension of the relationship between migration and MHCSU using a nationally representative cross-sectional dataset. The findings revealed that the likelihood of delivering birth at a health center was higher among the types of migration streams in comparison to the rural non-migrants, net of all control variables. The study revealed broadly comparable results concerning the utilization of at least four antenatal care (ANC) services, with two distinct trends. Firstly, the positive effect of all migration streams on four-plus ANC utilization was generally smaller in magnitude than institutional delivery care, and secondly, individuals who migrated from rural-to-rural areas exhibited lower likelihoods of seeking ≥4 ANC services than the rural non-migrants although this finding is not statistically significant in the fully adjusted model.

These findings align with conclusions from Thapa et al. (7) in Nepal, who also found that migration increased the probability of utilizing maternal healthcare services compared to rural non-migrants, and that the magnitude of this positive effect was higher for institutional delivery than for ≥4 ANC utilization (7). Our study also suggested that migration involving urban areas (to, from, or within) offered distinct advantages over purely rural migration streams. There appeared to be a notable advantage for women who migrated to or reside in urban areas, as well as those who move from urban areas, as opposed to their rural counterparts. This finding also found consistent in line with the study from Nepal (7).

After accounting for potential indicators of migrant selectivity in Model 2, the findings indicated that the estimated effect of migration streams on institutional delivery was reduced across all categories compared to Model 1. Furthermore, the effects became statistically insignificant for migration originating from rural areas (both rural-to-rural and rural-to-urban streams). A similar pattern was also observed for ≥4 ANC utilization, where after adjusting for selection factors, only urban-to-urban migrants were still significantly more likely to utilize services than rural non-migrants; the associations for all the other categories of migrants become insignificant.

This observed reduction in effect size after controlling for selection factors suggested that migrant selectivity likely played a significant role in the initial association observed between migration and MHCSU in Model 1. Thus, one reason for the higher likelihood of service utilization initially observed across migrant streams compared to rural non-migrants could be inferred: the migrant population may have possessed certain attributes (e.g., higher education, greater wealth, more autonomy) that made them more predisposed to utilizing maternal healthcare services, independent of the migration experience itself. However, it is important to note that even after adjustments, urban non-migrants and urban-to-urban migrants often remained significantly more likely to utilize services compared to rural non-migrants, aligning with results reported by Cotton in an African country context (22).

In the fully adjusted Model 3, the effect of the migration stream was further reduced for all of the categories of migration streams compared to Model 2 and becomes insignificant for migrants from urban to rural areas. Almost no difference was observed in the findings of ANC utilization between the second and third models. Even after controlling for the factors that affect maternal healthcare services utilization, urban-to-urban and urban non-migrants still had more likelihood of utilizing ≥4 ANC and institutional delivery compared to rural non-migrants.

Thus, following a meticulous inquiry into the effect of migration on MHCSU, while controlling for various confounding variables through sequential regression models, the findings of this study established a significant association between migration and the MHCSU. However, there was a remarkable trend within categories of migration streams while exerting its effect on maternal healthcare services utilization. Migration had a significant and positive association with MHCSU only in two scenarios: when migrating from an urban to a rural area (as in model 2), and when relocating between urban areas (as in model 3).

The superiority of urban migrants in terms of MHCSU is also reflected in studies in other country contexts (5, 23). This may be explained partly by an independent ‘urban effect’ on MHCSU, potentially related to the greater availability and accessibility of healthcare facilities and skilled medical professionals in urban destinations, irrespective of migrant selectivity. Migrants residing in or moving between urban areas may be more likely to access and utilize available healthcare services compared to those in rural areas. This suggests that the migrants in urban areas enjoy the ‘urban advantage’ in healthcare provision, which may drive the differential utilization of healthcare between urban and rural migrants.

The finding that urban-to-urban migrants had consistently high odds of MHCSU in both Model 2 and Model 3 may indicate that this group performed particularly well compared to rural non-migrants. However, it should be noted that these odds were calculated with rural non-migrants as the reference group. When comparing descriptive statistics or considering the odds ratios relative to urban non-migrants (who often served as the group with the highest utilization), the utilization level for urban-to-urban migrants typically fell short of that observed among urban non-migrants.

These findings align with previous research on migration and MHCSU, which often finds that among migrant groups, those migrating between urban areas tend to have high odds of utilization. However, similar to the findings of this study, these studies also reported that urban non-migrants have the highest likelihood of utilizing maternal healthcare services (5, 22–25).

The urban advantage in healthcare access is the likely underlying reason behind this. In addition, our bivariate analysis revealed that, within our sample, urban non-migrants exhibited the highest proportions with secondary or higher education, the greatest exposure to mass media, the highest levels of decision-making autonomy, and faced the fewest reported barriers (cost and distance) to accessing healthcare. These advantageous characteristics almost certainly benefited urban non-migrants, contributing to their higher maternal healthcare utilization levels.

There are some limitations exist in this study which need to be taken into consideration when interpreting the results. This study used data from BDHS, 2017–18 which adopts a cross-sectional design that may not be capable of establishing a causal relationship between migration and MHCSU. Consequently, formal testing of migrant selectivity, disruption, and adaptability processes over time was also not feasible. Additionally, reliably sequencing events like migration relative to specific pregnancies was challenging, as the BDHS did not inquire about the precise timing (year or month) of migration events.

## 5 Conclusion

This study provided an analysis of the implications of internal migration on maternal healthcare services utilization in Bangladesh. The research addressed a significant gap by identifying diverse migration streams and applying a rigorous analytical methodology to discern their independent effects on MHCSU. This approach yields a comprehensive understanding of the interplay between migration and maternal healthcare service utilization, thereby contributing substantially to the extant literature on this subject. The findings indicated that MHCSU varied notably between migrant and non-migrant groups, with the lowest access observed among rural non-migrant women. In contrast, migration to urban areas appeared to confer certain advantages in accessing and using maternal healthcare services. However, it was noteworthy that all migrant categories, regardless of their origin, exhibited lower use of maternal healthcare services compared to their urban non-migrant counterparts.

These insights underscore the necessity for future research to establish a causal link between migration and health outcomes and to delve deeper insights into the migration-health nexus. Based on our findings, a key policy recommendation is the development of public health strategies that explicitly consider migration status. Enhancing health information systems to capture more nuanced data on migrants’ needs and challenges is crucial. Such data can inform targeted, demand-specific programs. The outcomes of this study have significant implications for policy formulation and program development aimed at improving MHCSU for both migrant and non-migrant women in Bangladesh. Given the study’s finding that rural-to-rural migrants and rural non-migrants have lower utilization rates of maternal healthcare services compared to their urban counterparts, there is a pressing need to improve healthcare availability and accessibility, particularly in rural areas. Furthermore, the distinct challenges potentially confronted by migrants may necessitate healthcare programs tailored to their unique situations (e.g., considering integration challenges, portability of services). Such specialized programs would not only address the immediate healthcare needs of these populations but also contribute to the overarching aim of equitable healthcare access, thus supporting the broader goals of national development and the realization of sustainable development objectives.

## Data Availability

Publicly available datasets were analyzed in this study. This data can be found at: https://dhsprogram.com/data/available-datasets.cfm.

## Footnotes

^1^ https://dhsprogram.com/data/new-user-registration.cfm
